# The Antimicrobial Effect and ROS Redox Activity of Nb_2_O_5_-Containing Powders Obtained by the Sol–Gel Method

**DOI:** 10.3390/gels11090716

**Published:** 2025-09-07

**Authors:** Kalina Ivanova, Elitsa Pavlova, Iliana Ivanova, Albena Bachvarova-Nedelcheva

**Affiliations:** 1 Institute of General and Inorganic Chemistry, Bulgarian Academy of Sciences, Acad. G. Bonchev Str., Bl. 11, 1113 Sofia, Bulgaria; 2 National Centre of Excellence Mechatronics and Clean Technologies, 8 bul., Kl. Ohridski, 1756 Sofia, Bulgaria; 3 Faculty of Physics, Sofia University “St. Kliment Ohridski”, 5 James Bourchier Blvd., 1164 Sofia, Bulgaria; elli_pavlova@abv.bg; 4 Center of Competence “Clean Technologies for Sustainable Environment—Water, Waste, Energy for Circular Economy”, 1000 Sofia, Bulgaria; 5 Faculty of Biology, Sofia University “St. Kliment Ohridski”, 8 Dragan Tsankov Blvd., 1164 Sofia, Bulgaria; iaivanova@biofac.uni-sofia.bg

**Keywords:** antimicrobial effect, sol–gel, nanosized, powders, ROS, luminescence

## Abstract

The aim of the present paper is to study the antimicrobial effects of Nb_2_O_5_-containing nanosized powders. A combination of inorganic [telluric acid (H_6_TeO_6_)] and organic [Ti(IV) n-butoxide, Nb(V) ethoxide (C_10_H_25_NbO_5_)] precursors was used to prepare gels. To allow for further hydrolysis, the gels were aged in air for a few days. The gels were amorphous, but at 600 °C the amorphous phase was absent, and only TiO_2_ (anatase) crystals were detected. The average crystallite size of TiO_2_ (anatase) was about 10 nm. The UV-Vis spectrum of the as-prepared gel showed red shifting in the cut-off region. The obtained nanopowders were evaluated for antimicrobial properties against *E. coli* ATCC 25922, *P. aeruginosa* ATCC 27853, *S. aureus* ATCC 25923, and *C. albicans* 18804. Among these, only *E. coli* was examined in combination with the antibiotic ciprofloxacin to assess whether there was a potential synergistic effect. The results showed that the material exhibited antibacterial activity against the abovementioned bacterial strains but not against *C. albicans*. In the case of *E. coli* combined with ciprofloxacin, a concentration-dependent enhancement in antibacterial activity was observed. The obtained samples can be considered as prospective materials for use as environmental catalysts. The newly synthesized nanocomposite showed a balancing, modulating, and neutralizing effect on the generation of ROS. The inhibitory effect was preserved in all tested model chemical systems at pH 7.4 (physiological), indicating potential biological applications in inflammatory and oxidation processes in vivo.

## 1. Introduction

One of the most urgent issues facing global health today is the development of antibiotic resistance, which jeopardizes the efficacy of life-saving drugs and undermines decades of progress in medicine. Innovative approaches to countering microbial threats are desperately needed, as the overuse and abuse of antibiotics have led to the unrelenting growth of drug-resistant diseases [1]. The emergence of microorganisms that can cause serious incurable infections is a growing global concern, and traditional antibiotics are becoming less and less effective in treating patients [2,3]. In light of this, the World Health Organization (WHO) has identified antimicrobial resistance (AMR) as a major public health issue [4]. It has been found that nanomaterials are examples of novel antimicrobial materials that present promising approaches to fighting infections [5]. Addressing this crisis requires a multidisciplinary approach that incorporates developments in materials science, microbiology, and public health.

The application of nanosized materials, whose distinct physicochemical properties give them potent antibacterial activities, is one promising strategy in this multidisciplinary approach [6]. To fully appreciate their potential influence, it is essential to understand their mechanisms of action. These nanoparticles can exert their effects through multiple pathways [7]. While the generation of reactive oxygen species (ROS) is a well-established mechanism, several additional pathways also contribute to their bacteriostatic and bactericidal activities. These mechanisms work synergistically to target microbial cells at multiple levels, thereby reducing the likelihood of resistance development. Nanoparticles can physically interact with bacterial membranes, causing structural deformation, increased permeability, or complete disruption [8]. This can be due to induced mechanical stress or electrostatic interactions between the membrane and the charged surface of the nanoparticle [9]. When the integrity of the membrane is compromised, the leakage of intracellular components and eventual cell death can occur [10]. Surface charge, morphology, crystallographic orientation, and particle size can all influence the degree of membrane damage [11]. Certain particles (e.g., Cu, Ag, and ZnO) can release metal ions into the surrounding environment. Enzyme inactivation, replication disruption, and interference with energy production result from the penetration of these ions into bacterial cells and their binding to thiol groups in proteins or deoxyribonucleic acid (DNA) [12]. Another mechanism is the production of ROS—including hydroxyl radicals, superoxide anion radicals, and hydrogen peroxide [7]. They can damage vital molecules such as proteins, nucleic acids, and lipids. Oxidative stress can destroy cellular functions and induce apoptosis [13]. By altering important enzyme pathways or reducing adenosine triphosphate (ATP), nanoparticles may potentially interfere with bacterial metabolism [14]. Furthermore, interactions with ribosomes and DNA might prevent protein synthesis and replication, which hinders the development and viability of bacteria [15]. Certain nanoparticles have the ability to enter or stop the development of biofilms, which are organized bacterial populations with a high level of resistance to antibiotics [16]. Nanoparticles provide a strong and versatile antibacterial strategy by directly attacking bacteria via a variety of methods. Size, shape, and surface chemistry are some of their adjustable characteristics that can increase their potential to be adaptable tools in next-generation antimicrobial therapies [14]. Because of their special qualities, metal nanoparticles stand out among the most promising innovations [17,18].

According to the literature review, the antibacterial properties of titanium (Ti), tellurium (Te), and niobium (Nb) have attracted scientific attention among the wide variety of metal nanoparticles under investigation [1,19,20]. Generally, most metal nanoparticles can efficiently destroy microbiological integrity because of their special physicochemical characteristics, which include high surface-area-to-volume ratios and variable chemical activity [18,21]. Their potential to combat AMR is highlighted by their modes of action, which include membrane rupture, oxidative stress, and interference with vital cellular processes [21,22]. Additionally, they are essential in the development of next-generation antimicrobial medicines due to their versatility for biofunctionalization and compatibility with current medical technologies [23].

In the study of Preda et al. [24], the antibacterial properties of TiO_2_ nanoparticles doped with Zn and Cu synthesized by the sol–gel method were reported. The powder samples were tested against the Gram-positive representative—*S. aureus*. The results showed a good inhibition rate of 85.47% for TiO_2_-Cu and 84.85% for TiO_2_-Zn powders. In the work of Priyanka et al. [25], the antimicrobial properties of calcinated TiO_2_ nanoparticles synthesized by the sol–gel method were investigated. They tested various microorganisms, representatives of both Gram-positive (*Streptococcus pneumoniae*, *Staphylococcus aureus*, and *Bacillus subtilis*) and Gram-negative bacteria (*Proteus vulgaris, Pseudomonas aeruginosa,* and *Escherichia coli*) and pathogenic yeast (*Candida albicans*). It could be generalized that TiO_2_ (rutile) had sensitivity only for some bacterial strains (*S. pneumoniae, S. aureus, P. aeruginosa*, and *Candida albicans*), while TiO_2_ (anatase) showed significant antimicrobial activity in comparison to rutile. This could be explained by the fact that calcinated forms of TiO_2_ can vary in particle size, which may result in reduced antimicrobial activity [25].

Despite their great toxicity, tellurites have been used to treat a number of illnesses, including leprosy, syphilis, tuberculosis, dermatitis, cystitis, and eye infections. Thus, before the invention of antibiotics, soluble salts containing Te were employed as antibacterial and therapeutic agents [26]. Regardless of the degree of resistance, tellurite groups disrupt the transmembrane proton gradient in *E. coli* cells upon entry. During aerobic expansion, this action is coupled with the suppression of ATP synthesis, which causes the intracellular ATP stores to be depleted [27]. Ao et al. [28] evaluated the antibacterial properties of biosynthesized tellurium nanoparticles using *Moringa oleifera* extract. The bio-Te nanoparticles exhibited good antibacterial activity based on the results of inhibition zone tests against *Escherichia coli*, *Salmonella typhimurium*, *Klebsiella pneumoniae*, *Shigella dysenteriae*, *Streptococcus agalactiae*, and *Streptococcus pneumoniae* [28,29].

Niobium is thought to function as one of the so-called “essential metals”, similar to titanium, due to its chemical stability within the physiological state [29]. Because of their excellent chemical stability and tolerable biocompatibility, niobium and niobium alloys are widely used in biomaterials [30,31].

Recently, it has been reported that TiO_2_-based powders containing B_2_O_3_ and Nb_2_O_5_ exhibit antibacterial properties against *E. coli* NBIMCC K12 407 and *B. subtilis* NBIMCC 3562 [32]. Gram-positive bacteria were inhibited but generally required higher concentrations or longer exposure. On the other hand, Souza et al. [33] evaluated the antibacterial activity of composites containing niobium pentoxide (Nb_2_O_5_) and titanium dioxide co-doped with fluorine and nitrogen. They found that the composites containing 2% niobium pentoxide significantly reduced the formation of *S. mutans* biofilms. According to Boruah et al. [34], Nb_2_O_5_ nanoparticles synthesized via the solution combustion technique and doped with Sr, Y, Zr, and Ag exhibited antibacterial properties. The Ag, Zr, and Sr-doped Nb_2_O_5_ nanoparticles showed a 1.5 log reduction in the number of bacterial cells for *E. coli*, and a 3-log reduction was observed from Y-doped Nb_2_O_5_. For the Gram-positive representative, *S. aureus*, the bacterial cell reduction was observed around 0.5 log (pristine Nb_2_O_5_) and a maximum of 2 log-fold from Sr doped Nb_2_O_5_.

If metal nanoparticles are utilized in biological systems, they could interact with metabolites and cellular structures. It is critical to evaluate their safety or, on the opposite, their utility as antibacterial and cytotoxic compounds for use in a variety of practical applications. Free radical reactions and ROS production are essential in homeostasis and ensure the organism’s metabolic equilibrium, functional activity, and adaptation. These processes and interactions are researched when testing drugs, food extractions and additives, nanomaterials safety, and other biological effects [35,36,37].

This study aims to verify the antimicrobial properties of Nb_2_O_5_-containing powders synthesized by the sol–gel method. Additionally, the investigations show that it is feasible to combine nanostructures and traditional antibiotics in ways that are efficient in preventing the development of bacterial biofilms and bacterial resistance. Furthermore, there is a noticeable lack of research addressing the synergistic effects of incorporating titanium, niobium, and tellurium within such nanostructures. The results will be helpful in determining how Nb_2_O_5_ and the selected composition affect the properties of the newly synthesized material, which will lead to new discoveries in the fields of environmental technologies and in vivo applications.

## 2. Results and Discussion

### 2.1. XRD and SEM-EDX Investigations of the Powders

**Figure 1:**
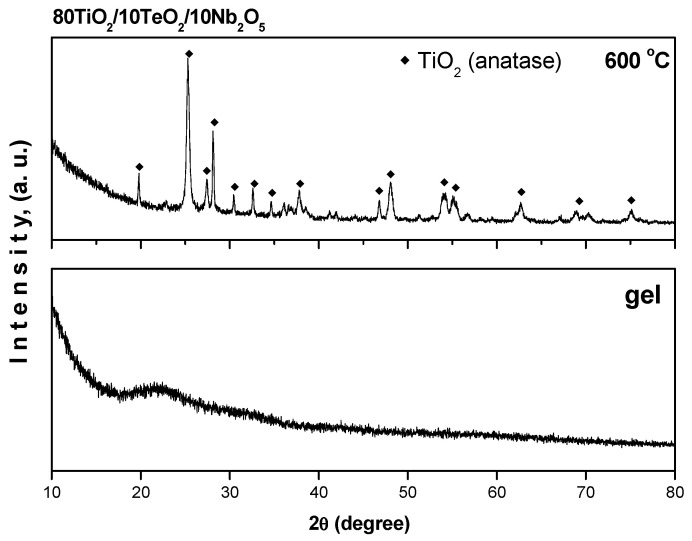
XRD patterns of the investigated sample 80TiO_2_/10TeO_2_/10Nb_2_O_5_.

XRD was used to examine the crystallinity and phase development of the examined ternary sample that had been calcined at 600 °C. Figure 1 displays the samples’ XRD patterns. As is seen from the figure, the amorphous gel is completely converted into crystalline after heating at 600 °C for a 2 h exposure time. The TiO_2_ (anatase, JCPDS 78-2486) crystals were detected only at this temperature. In general, the XRD patterns structurally point out that the obtained powders are highly pure due to the absence of undesired impurity diffraction peaks generated from the precursors used for the synthesis.

To analyze the crystallite growth, the crystallite size of the TiO_2_ nanoparticles was calculated by the Scherrer equation. Using the strongest (101 peak), it was established that the crystallite size is about 25 ± 0.5 nm. The obtained XRD data are very similar to those reported in other papers [37,38]. The BET measurements established that the specific surface area (SSA) of the investigated samples is about 35–45 m^2^/g.

#### Electron Microscopy and EDX Analysis

**Figure 2:**
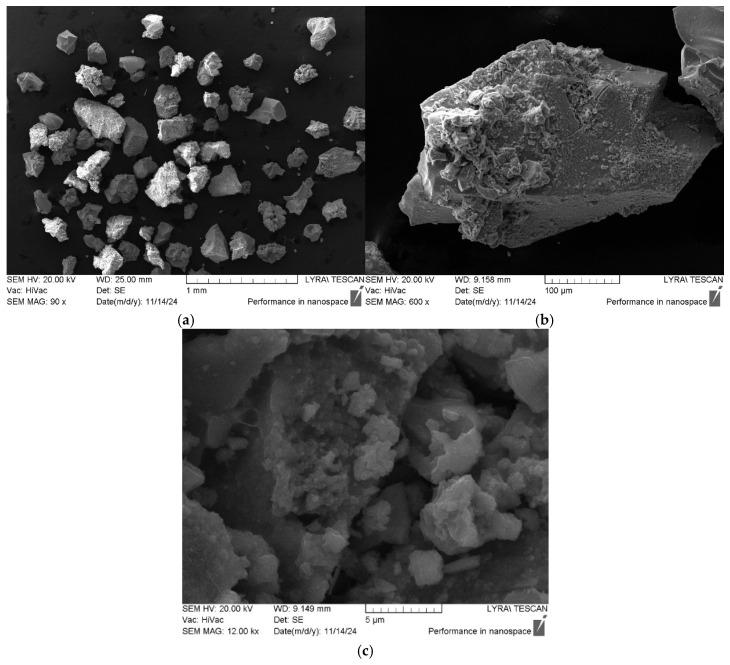
SEM images of 80TiO_2_/10TeO_2_/10Nb_2_O_5_ sample heated at 600 °C taken at different magnifications: ×90 (**a**), ×600 (**b**), and ×12,000 (**c**).

**Figure 3:**
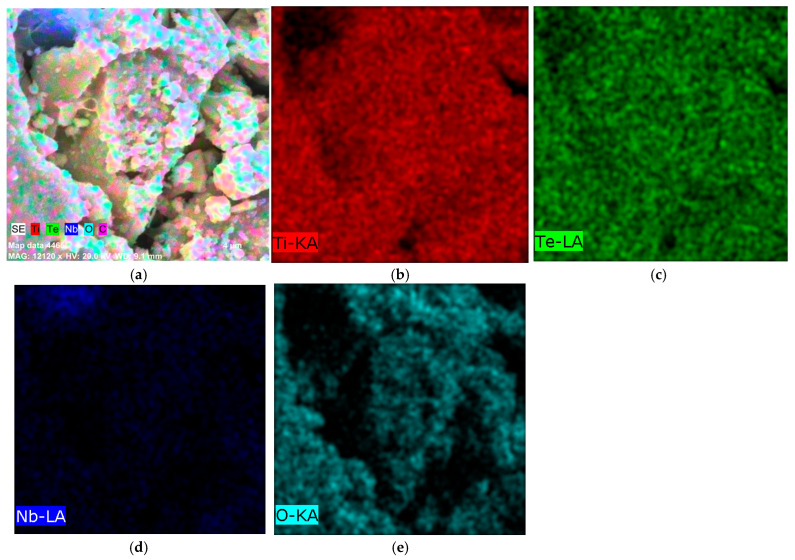
SEM image elemental mapping of the investigated sample (**a**); composition map of Ti (**b**); composition map of Te (**c**); composition map of Nb (**d**); composition map of O (**e**).

The morphology and material composition obtained by the sol–gel method were verified by SEM (Figure 2a,c) and EDX spectroscopy (Figure 3a–e). The SEM micrographs of the heat-treated sample (Figure 2a–c) showed a structure of the particles consisting of well-defined shapes and agglomerated grains varying both in morphology and particle size above 1 μm. The observed particle agglomeration is typical for the sol–gel-derived materials, and it is probably due to the high surface peculiarities and the calcination temperature. Similar results for the N_2_O_5_-containing powders have been obtained by other authors as well [39].

**Figure 4:**
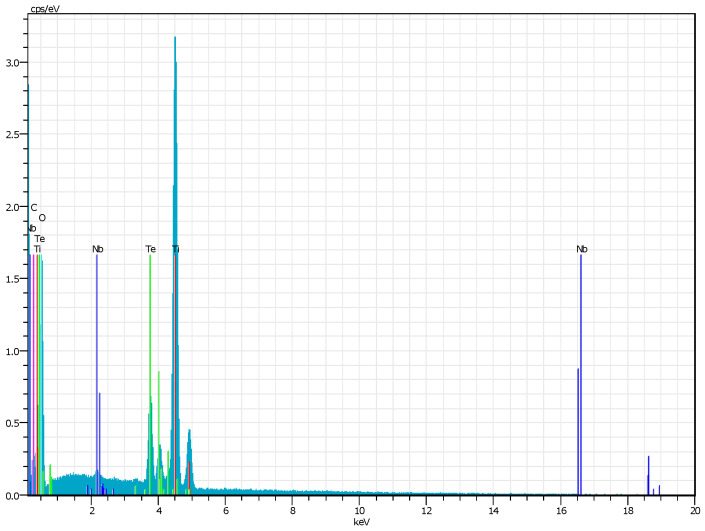
EDX results for the investigated sample.

The corresponding energy dispersive X-ray (EDX) elemental mapping (Figure 3a–e, Figure 4) confirmed the presence of Ti, Te, Nb, and O as the main elements. No impurities were detected, which indicates the successful synthesis and high purity of the material. A homogeneous distribution of Ti, Te, and O elements was observed as well. Additionally, elemental mapping demonstrated that Nb and Te were implying successful integration into the TiO_2_ matrix during the sol–gel process and subsequent thermal treatment at 600 °C. Uneven distribution was observed for Nb (Figure 3d). Moreover, the obtained results revealed that powders prepared via the sol–gel method exhibited aggregated nanoparticles and interconnected microstructures after thermal treatment. The observed morphological peculiarities arise from variations in condensation during solvent removal, demonstrating how drying dynamics control homogeneity [40,41]. The EDX analysis correlate well with the results obtained by other authors [42,43].

### 2.2. IR and UV-Vis Spectroscopy Results

**Figure 5:**
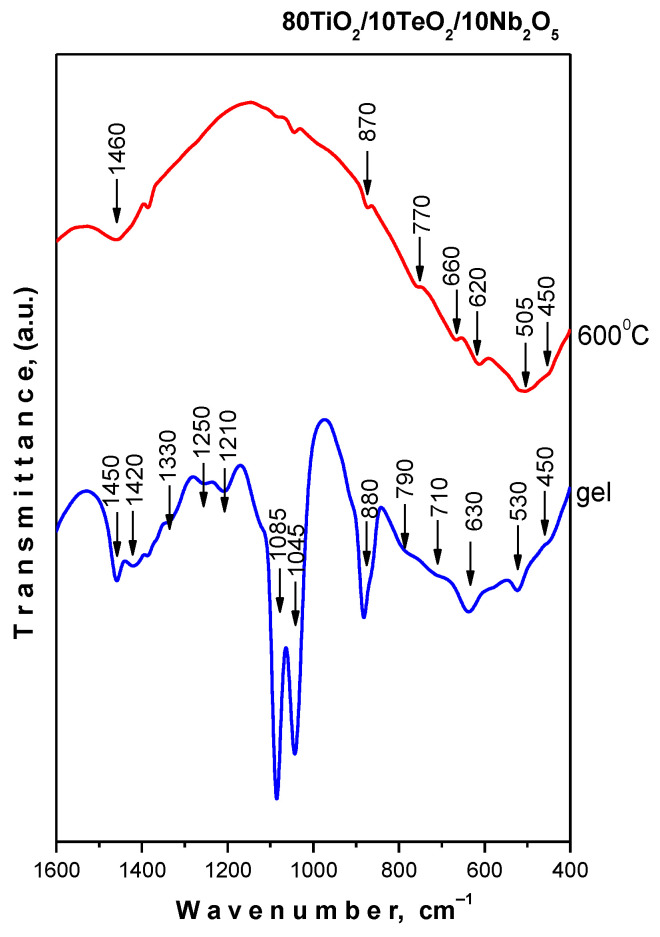
IR spectra of the gel and sample heated at 600 °C.

IR spectra of the gel and samples heated at 600 °C are shown in Figure 5. Spectral data from our earlier studies on sol–gel-generated binary and ternary composite powders, including TiO_2_, were used to assign the vibration bands [44,45,46]. The gel exhibited the presence of bands at 1120, 1090–1080, and 1040–1030 cm^−1^ that could be attributed to the Ti-O-C stretching vibrations creating the formation of a mixed organic–inorganic amorphous structure. On the other hand, the bending vibrations of CH_3_ and CH_2_ groups are also situated in this region [47,48].

The spectral behavior of the sample at 600 °C showed vibrations of inorganic structural units only. These bands are with low intensity and broadened, which is typical for the disordered systems. The observed bands in the region 700–400 cm^−1^ are related to the vibrations of a Ti-O-Ti network [49,50]. The observed strong bands at 670 and 620 cm^−1^ are assigned to the vibrations of TeO_4_ trigonal bipyramids (tbp) [49,50]. There is strong overlapping in the 750–400 cm^−1^ region, which hinders the more precise assignments of the inorganic bands. The infrared band at 870 cm^−1^ can be ascribed to the ν_1_ stretching vibration of short Nb-O bonds in isolated NbO_6_ octahedra [51,52]. The acquired results are in good agreement with the previously described XRD data and our previous studies on the sol–gel production of compositions containing TiO_2_ [44,45,47,48,49,50,51,52].

**Figure 6:**
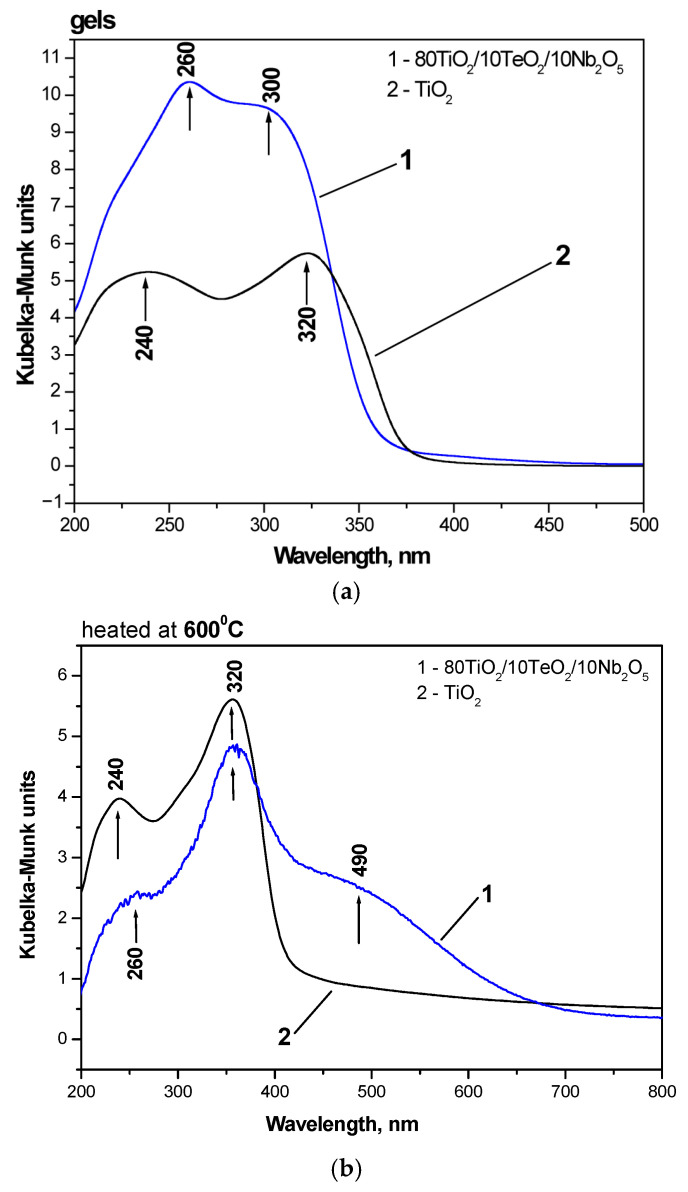
UV-Vis spectra of the investigated samples compared with Ti(IV) butoxide: gels (**a**) and powders heat-treated at 600 °C (**b**).

In Figure 6, the optical absorption spectra of the investigated gel compositions are compared to those of TiO_2_ obtained by Ti(IV) butoxide. The UV-Vis spectra of the heat-treated samples are presented in Figure 6b. Both gels possessed good absorption in the UV region, where the ternary sample 80TiO_2_/10TeO_2_/10Nb_2_O_5_ exhibited the highest absorption.

The isolated TiO_4_ and TiO_6_ units were responsible for the two peaks in the UV-Vis spectra of both samples that were observed at 260 and 300 nm, respectively [53]. Comparable UV peak intensities at 240 and 320 nm were seen in the TiO_2_ gel, indicating that there were equivalent amounts of TiO_4_ and TiO_6_ polyhedra in the gel network. The UV-Vis spectra of the 80TiO_2_/10TeO_2_/10Nb_2_O_5_ gel exhibited that the band at about 260 nm was with higher intensity than that centered at 300 nm. This suggests that the unhydrolyzed sample is dominated by TiO_4_ groups.

The optical bandgap (Eg) of the studied samples was also ascertained using the UV-Vis spectra. The absorption edge values of the Nb_2_O_5_-containing gel were 363.2 nm in both absorption spectra, whereas the absorption edge values of pure TiO_2_ were 389.71 nm. When compared to the pure TiO_2_ gel, this indicates a blue shift for the studied material. As stated in the literature [54,55,56], the calculated bandgaps of the examined gels are 3.41 and 3.18 eV, respectively. As a typical n-type wide-bandgap semiconductor, pure Nb_2_O_5_ has a bandgap of 3.4 eV, according to the literature, and the results obtained are extremely near that value [57]. The wavelength and intensity of the Nb_2_O_5_ absorption spectra are dependent on the size, morphology, crystalline type, and synthesis process, according to certain scientists. Blue shifting in the absorption spectra is caused by the reduced size of Nb_2_O_5_ particles [55,58].

As is seen from Figure 6b, after heat treatment at 600 °C, the UV-Vis spectra shapes have been changed. As a result of the hydrolysis–condensation processes, the coordination geometry is changed to TiO_6_ as a result of polymerized Ti species (Ti–O–Ti links between TiO_6_ units). An indication for this phenomenon is the increased intensity of the band at 320 nm related to the higher amount of TiO_6_ groups. The other peculiarity is the redshift of the absorption edge in the spectra of the ternary sample (Table 1). These findings align with the literature reporting on Te- and C-modified TiO_2_ compositions [59,60]. The development of an impurity energy level within the TiO_2_ bandgap was cited as the explanation for this behavior [59,60,61]. Carbon was found to be another strong factor responsible for the visible light absorption in the 490 nm [62].

### 2.3. Antimicrobial Property Results

In this study, we investigated the antimicrobial activity of Ti/Te/Nb hybrid nanopowders both alone and in combination with ciprofloxacin. The tested microorganisms included the Gram-positive bacteria *Staphylococcus aureus* ATCC 25923, the Gram-negative bacteria *Escherichia coli* ATCC 25922 and *Pseudomonas aeruginosa* ATCC 27853, and the yeast *Candida albicans* ATCC 18804. Among these, only *E. coli* was examined in combination with an antibiotic to evaluate a potential synergistic effect, as it showed the lowest bactericidal concentration when treated with the nanopowders alone.

Two types of tests—qualitative (spot test) and quantitative (microdilution method)—were carried out to obtain more comprehensive data regarding the antibacterial activity of the prepared sample.

**Figure 7:**
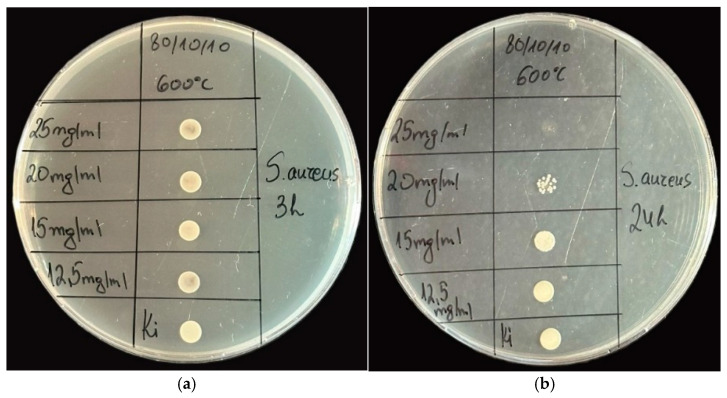
Spot test of sample 80TiO_2_/10TeO_2_/10Nb_2_O_5_ at the 3rd (**a**) and 24th (**b**) hours evaluating the antibacterial effect against *S. aureus*.

**Figure 8:**
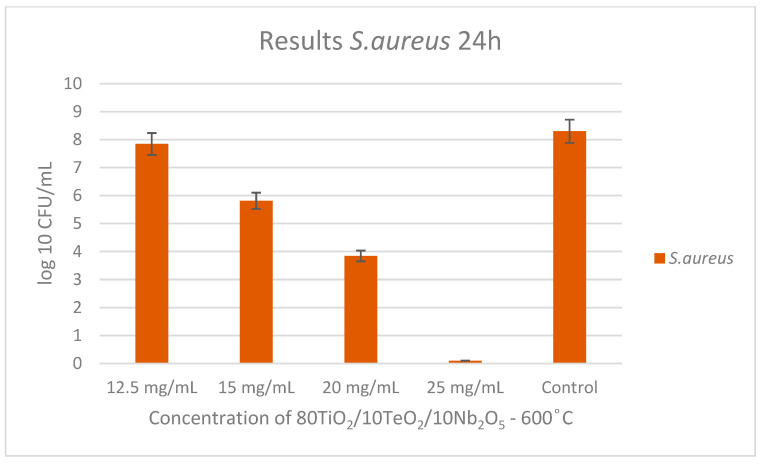
Antibacterial effect of 80TiO_2_/10TeO_2_/10Nb_2_O_5_ heat-treated at 600 °C tested at different concentrations against *S. aureus*.

As shown in Figure 7 and Figure 8, the ternary sample heated at 600 °C showed antibacterial activity, and the MIC and MBC were determined. For the Gram-positive bacteria *S. aureus,* the MBC was determined to be 25 mg/mL, and the MIC was 20 mg/mL at the 24th hour. No inhibition was observed at the 3rd hour with the concentrations of nanopowders used.

**Figure 9:**
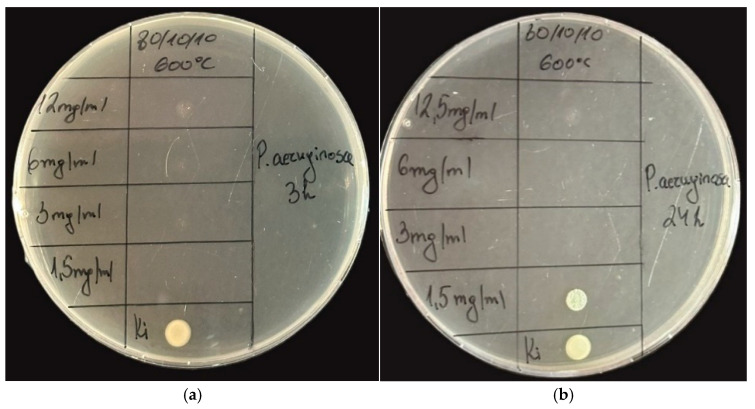
Spot test of sample 80TiO_2_/10TeO_2_/10Nb_2_O_5_ at the (**a**) 3rd and (**b**) 24th hours evaluating the antibacterial effect against *P. aeruginosa*.

**Figure 10:**
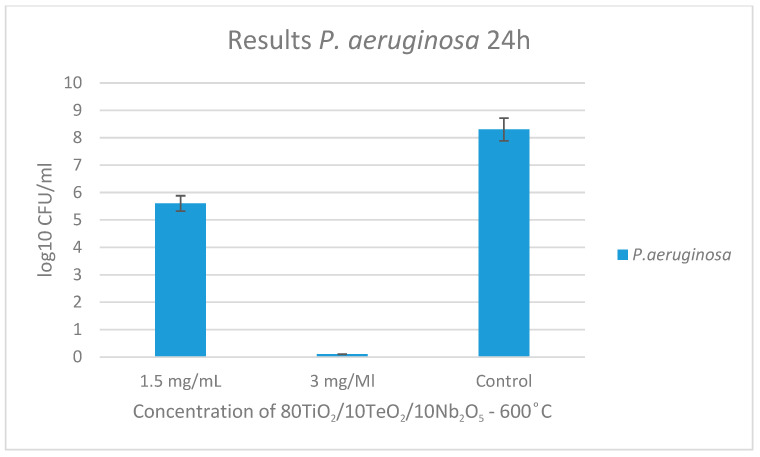
Antibacterial effect of 80TiO_2_/10Nb_2_O_5_/10TeO_2_ heat-treated at 600 °C and tested at different concentrations against *P. aeruginosa*.

The results of the tested sample and its antibacterial activity against the Gram-negative pathogen *Pseudomonas aeruginosa* are presented in Figure 9 and Figure 10. At the 3rd-hour mark, no bacterial growth was observed except in the control sample. By the 24th hour, the MIC was determined to be 1.5 mg/mL, while the MBC was established to be 3 mg/mL.

**Figure 11:**
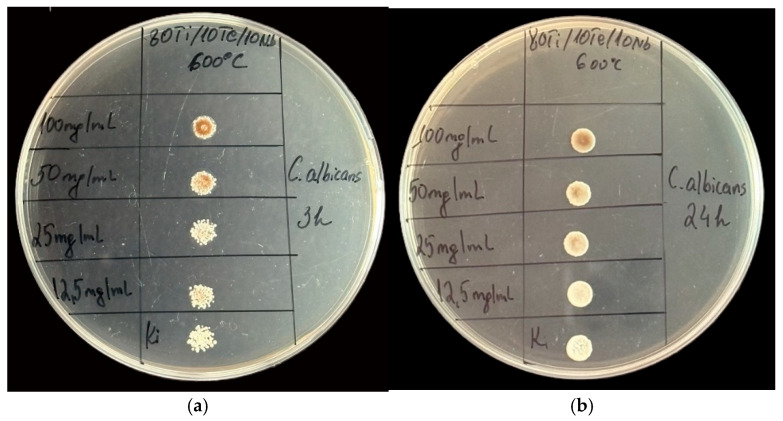
Spot test of the 80TiO_2_/10Nb_2_O_5_/10TeO_2_ sample at the 3rd (**a**) and 24th hours (**b**) evaluating the antifungal effect against *C. albicans*.

The synthesized material was tested also on *Candida albicans*, but there was not any fungicidal effect, as shown in Figure 11.

**Figure 12:**
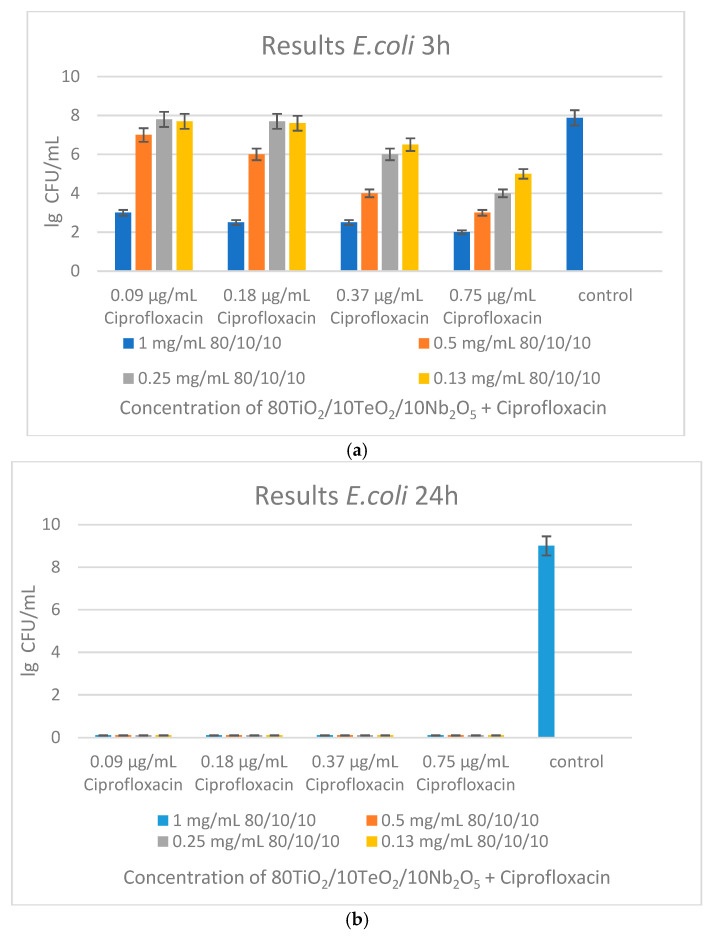

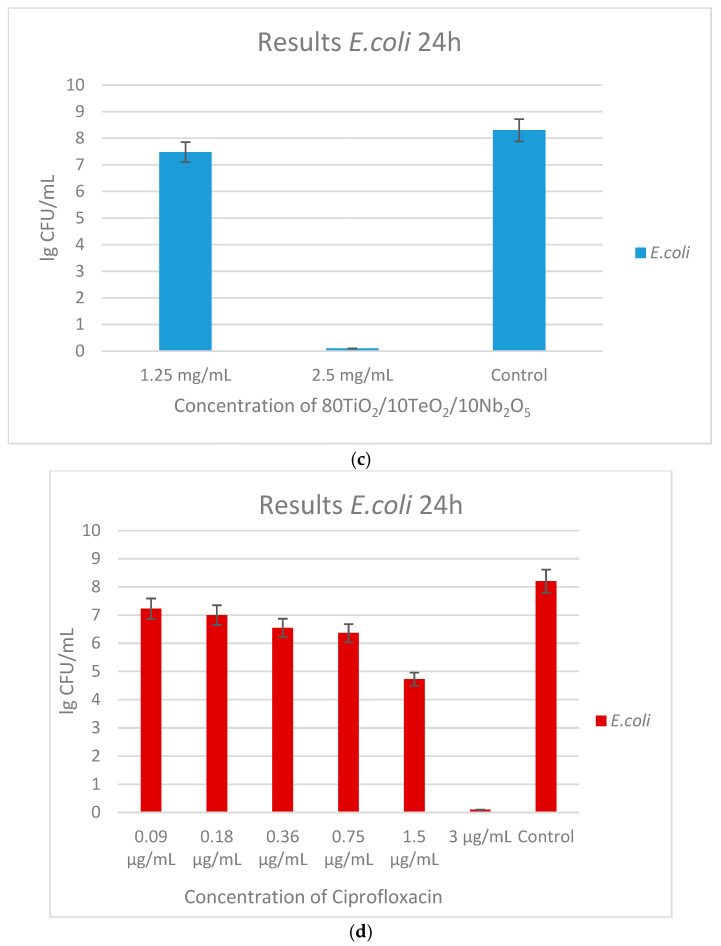
Antibacterial effect of (**a**) 80TiO_2_/10TeO_2_/10Nb_2_O_5_ heat-treated at 600 °C with ciprofloxacin at the 3rd hour; (**b**) 80TiO_2_/10TeO_2_/10Nb_2_O_5_ heat-treated at 600 °C with ciprofloxacin at the 24th hour; (**c**) 80TiO_2_/10TeO_2_/10Nb_2_O_5_ heat-treated at 600 °C only; (**d**) ciprofloxacin only.

The antibacterial efficacy of the sample in combination with ciprofloxacin was evaluated against *E. coli* at the 3rd and 24th hours (Figure 12a,b). At the 3rd hour, partial inhibition of bacterial growth was observed in all concentrations of the nanopowder (0.13–1 mg/mL) when combined with varying concentrations of ciprofloxacin (0.09–0.75 µg/mL), indicating early suppressive activity. However, full bactericidal activity was achieved at the 24-h mark. All nanoparticle–ciprofloxacin combinations resulted in the total eradication of *E. coli* independent of the concentrations used, while the control group remained viable (Figure 12a,b).

Individual treatments were evaluated in order to further distinguish the contributions of each agent (Figure 12c,d). The powdered material alone reduced colony counts moderately at 1.25 mg/mL, while at 2.5 mg/mL it showed full eradication of bacteria, indicating a dose-dependent effect. Ciprofloxacin alone showed a gradual, dose-dependent bactericidal effect, with complete inhibition at 3 µg/mL These findings suggest a strong synergistic effect between ciprofloxacin and the nanopowders, enabling bacterial elimination at significantly lower antibiotic concentrations than when used alone. This highlights the potential of the sample to improve antibiotic performance and reduce the dosage needed.

It could be generalized that the Nb_2_O_5_-containing nanopowders exhibited measurable antibacterial activity against both Gram-positive *S. aureus* ATCC 25923 and Gram-negative *E. coli* ATCC 25922 and *P. aeruginosa* ATCC 27853, with the effect being notably stronger against the Gram-negative strain, as indicated by its lower MIC (1.25; 1.5 mg/mL) and MBC (2.5; 3 mg/mL) values compared to those for *S. aureus* (MIC 20 mg/mL; MBC 25 mg/mL). However, the sample showed no antifungal activity against *C. albicans* ATCC 18804, indicating a selective and enhanced antibacterial efficacy primarily targeting Gram-negative pathogens.

### 2.4. Chemiluminescent ROS Redox Activity Tests

When using metal nanomaterials in living systems, one should always have in mind that these often have completely different properties and behavior compared to the same material in its macro-dimensions. Therefore, it is necessary to study and describe the properties of all newly created nanomaterials, including their redox activity and effects on the free radical generation reactions. These processes and ROS are essential for metabolism and homeostasis. They can be evaluated by the activated chemiluminescent method.

Chemiluminescence allows the monitoring of ROS concentrations and kinetics of free radical reactions in the range of 480–580 nm. It can easily describe the pro-oxidant/antioxidant (inhibitory) activity of the tested material, which harm or have a positive impact on a living organism, respectively. The extremely weak signal obtained from these reactions can be amplified many times with the help of physical and chemical activators (probes). One that we have also applied is lucigenin. This method allows the automatization and registration of the kinetics of the reactions, the valuation of rate constants, and other parameters of the interaction of the studied substance.

In the present work, a newly synthesized composite containing titanium, tellurium, and niobium oxides was studied at a concentration of 1 mg/mL. Its effect on model reactions of free radical oxidation was evaluated. All their products are normally formed metabolites; their hyper-concentrations lead to oxidative stress and are generated during inflammation, immune response, or the progression of various diseases. The recorded signal was compared to a control reaction that did not contain the studied nanomaterial (blank control).

**Figure 13:**
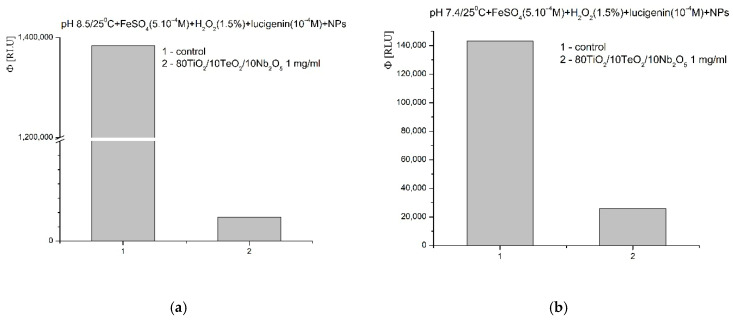
Effect of Nb_2_O_5_-containing nanosized powders on chemiluminescence, presented as quantum yields, in a system for the generation of ^·^OH and ^·^OOH radicals at pH 8.5 (**a**) and pH 7.4 (**b**).

In Fenton’s model system ^·^OH and ^·^OOH radicals are generated. A pH of 8.5 is optimal for the reaction; the newly synthesized nanomaterial showed very strong inhibitory activity towards the generated radicals—almost 30-fold inhibition of the reaction and the signal, compared to the blank control (Figure 13a). At pH 7.4 (physiological), a reaction-suppressing effect was also observed, ~80%, compared to the control reaction (Figure 13b).

**Figure 14:**
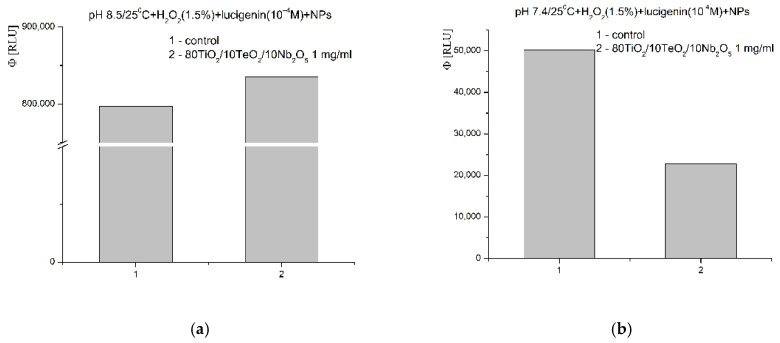
Effect of Nb_2_O_5_-containing nanosized powders on chemiluminescence, presented as quantum yields, with oxidant H_2_O_2_ at pH 8.5 (**a**) and pH 7.4 (**b**).

In the system containing the strong oxidant hydrogen peroxide (H_2_O_2_), at pH 8.5, a weak pro-oxidant activity and stimulation of the oxidation reaction was observed. The significant increase in the luminescent signal was ~5% when compared to the control reaction (Figure 14a). This effect was not preserved at pH 7.4. Towards that ROS, which is a signaling molecule and a molecule associated with the immune response in the body, the newly synthesized nanomaterial showed a pronounced inhibitory activity. The effect was more than 55% compared to the control (Figure 14b).

**Figure 15:**
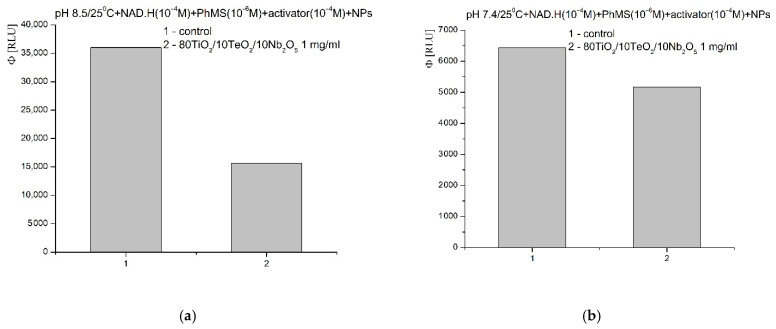
Nb_2_O_5_ containing nanosized powders on chemiluminescence, presented as quantum yields, in a system for the generation of O_2_^−.^ radicals at pH 8.5 (**a**) and pH 7.4 (**b**).

In the model chemical system for the generation of O_2_^−.^ radicals, at pH 8.5, the nanomaterial presented a similar inhibitory activity. The effect was over 55% compared to the control (Figure 15a). The observed inhibition effect was maintained at pH 7.4—about a 20% lower signal compared to the blank reaction (Figure 15b).

Based on the results obtained, the following effects can be described, and a working hypothesis can be built for the mechanism of inhibitory action of the newly synthesized nanomaterial containing titanium, tellurium, and niobium oxides with respect to its redox activity towards free radicals and ROS:

- It is possible that the sorption and stabilization of free radicals and ROS can take place on the surface of the nanocomposite—the oxides of titanium, tellurium, and niobium can probably act as surface traps for ROS, preventing their reactivity in the environment; their large mass, on the other hand, probably leads to rapid precipitation and hinders the reactions in an aqueous solution;
- It is possible that the catalytic degradation or transformation of ROS results in less reactive forms when the nanomaterial participates as a redox-active surface, especially at a higher pH 8.5, when the observed effects were most pronounced;
- Most likely, the newly synthesized material has selective, extremely weak pro-oxidant activity towards H_2_O_2_; this activity was registered only in weak, basic media (pH 8.5).

In summary, the newly synthesized nanocomposite demonstrated a balancing, modulating, and neutralizing effect on the generation of ROS. The preservation of the inhibitory effect in all modeled chemical systems at a physiological pH of 7.4 indicates its potential biological application (e.g., in inflammatory and oxidation processes in an organism). The synergy between Ti, Te, and Nb may be responsible for the specific inhibitory effect on the different types of free radicals and ROS generated in extremely high concentrations during inflammation, immune response, and disease progression in the body.

## 3. Conclusions

Nb_2_O_5_-containing powders were obtained by applying the sol–gel method. According to the XRD after heat treatment at 600 °C, TiO_2_ (anatase) was identified without the presence of impurities. IR research revealed that at 600 °C, the organic–inorganic amorphous phase is transformed into an inorganic one. Two maxima associated with the isolated TiO_4_ units and condensed TiO_6_ groups were detected using UV-Vis spectroscopy at around 260 and 300 nm, respectively.

The antimicrobial effect of the TiO_2_/TeO_2_/Nb_2_O_5_ composition has been investigated in detail. The sample exhibited good antibacterial properties, with notably better efficacy against the Gram-negative strains *E. coli* ATCC 25922 and *P. aeruginosa* ATCC 27853 compared to the Gram-positive *S. aureus* ATCC 25923. Among the tested strains, *E. coli* demonstrated the highest sensitivity with MBC 2.5 mg/mL, which was improved even more when ciprofloxacin was added, indicating a definite concentration-dependent synergistic effect. However, when tested against *C. albicans*, the sample exhibited no significant antifungal activity. According to the results, the sample under investigation has the potential to be employed as an effective antibacterial agent, especially against Gram-negative bacteria, and supports further investigation into their mechanism and clinical applications.

The newly synthesized nanocomposite showed a balancing, modulating, and neutralizing effect on the generation of ROS. The inhibitory effect was preserved in all tested model chemical systems at pH 7.4 (physiological), indicating potential biological applications in inflammatory and oxidation processes in vivo.

In conclusion, the TiO_2_/TeO_2_/Nb_2_O_5_ nanocomposite demonstrated promising physicochemical characteristics and biological activity, including notable redox-modulating and antibacterial properties, especially against Gram-negative bacteria like *E. coli*, with enhanced efficacy when combined with ciprofloxacin. These results support its potential for biomedical applications in managing oxidative stress and bacterial infections.

## 4. Materials and Methods

### 4.1. Gels Preparation

A sample with nominal composition 80TiO_2_/10TeO_2_/10Nb_2_O_5_ was subjected to a detailed investigation. The gel was synthesized by combining Te(VI) acid (99.99%, Aldrich, St. Louis, MO, USA) with Ti butoxide(IV) (≥99%, Fluka AG, Buchs, Switzerland) and niobium(V) ethoxide (C_10_H_25_NbO_5_) (Merck, Darmstadt, Germany) as precursors dissolved in ethylene glycol (C_2_H_6_O_2_) (99%, Aldrich, St. Louis, MO, USA). The problem of the high rate of hydrolysis of tellurium (VI) alkoxide, which has been examined in a number of articles [63,64], was addressed by the use of telluric acid (H_6_TeO_6_). The precursor solutions were vigorously stirred for 5–10 min at room temperature in order to ensure full dissolution. There was no direct addition of water to the precursor solutions. Absorbing ambient moisture was the source of the sol–gel hydrolysis reaction. Depending on the composition, the observed pH ranged from 4 to 5. For the compositions under investigation, the gelation happened right away. The gels were aged in air for a few days in order to finish the hydrolysis. The resulting gel was heated to 600 °C for two hours and then exposed to air until it turned into powder. Our earlier research served as the basis for choosing the temperature [63].

### 4.2. Samples Characterization

At room temperature, the powder XRD patterns were recorded with a Bruker D8 Advance (Berlin, Germany) X-ray powder diffractometer with Cu Ka radiation (k = 1.54056 Å) with a LynxEye solid position sensitive detector and X-ray tube operated at 40 kV and 40 mA. X-ray diffraction patterns were recorded in the range of 5.3–80° for 2 h with a step of 0.02° 2 h. The infrared spectra were made in the range 1600–400 cm^−1^ using the KBr pellet technique on a Nicolet-320 FTIR spectrometer (Madison, WI, USA) with 64 scans and a resolution of ±1 cm^−1^. The samples were photographed by a scanning electron microscope (SEM) JSM-5510 (JEOL Ltd., Tokyo, Japan) operated at a 10 kV of acceleration voltage. The investigated samples were coated with carbon by JFC-1200 fine coater (JEOL, USA, Inc., Peabody, MA, USA) before observation. The energy dispersive X-ray spectroscopy (EDS) analysis was carried out on a Zeiss Evo 15 microscope (Bruker Resolution 126 eV, Berlin, Germany). A UV-Vis diffused reflectance spectrophotometer Evolution 300 (Thermo Electron Corporation, Madison, WI, USA) with a magnesium oxide reflectance standard as the baseline was used for recording the optical absorption spectra of the powdered samples in the wavelength range of 200–800 nm. To determine the absorption edge and optical bandgap, Planck’s equation was utilized (Eg) [65,66,67]. The BET equation was used to calculate the specific surface areas (BETs) of nitrogen adsorption at low temperatures (77.4 K) in a NOVA 1200e surface area and pore analyzer (Quantachrome, Boynton Beach, FL, USA) at relative pressures *p*/*p*0 = 0.1–0.3.

### 4.3. Antimicrobial Activity Testing

To comprehensively evaluate the antibacterial and antifungal properties of the sample under investigation, we employed a panel of four microbial strains: three bacterial species—*Staphylococcus aureus* ATCC 25923, *Pseudomonas aeruginosa* ATCC 27853 and *Escherichia coli* ATCC 25922—as well as a yeast representative—*Candida albicans* ATCC 18804. These particular strains were selected based on their clinical importance and the diversity of their cell wall structures, which include both Gram-positive (*S. aureus*) and Gram-negative (*P. aeruginosa, E. coli*) bacteria, in addition to a fungal pathogen.

To determine the antibacterial activity of the nanopowders, we performed a spot test assay at two specific time intervals—at the third and twenty-fourth hours post-inoculation. In parallel, the minimal inhibitory concentration (MIC) and minimal bactericidal concentration (MBC) were determined using the standardized broth microdilution method, which is a widely accepted quantitative approach for assessing antimicrobial potency [68].

Bacterial suspensions were prepared to match a turbidity equivalent to a 0.5 McFarland standard, which corresponds to an approximate cell density of 1.5 × 10^8^; a fungal suspension of 0.5 McFarland corresponds to 10^6^ CFU/mL. Antibacterial activity of the sample, alone and in combination with ciprofloxacin, was evaluated using the broth microdilution method in sterile 96-well plates. Ciprofloxacin was serially diluted two-fold, ranging from 100 µg/mL to 0.09 µg/mL. For combination testing, 50 µL of each antibiotic dilution was mixed with 50 µL of the nanopowder suspension in the corresponding concentration. Then, 100 µL of the prepared bacterial suspension was added to each well. For the testing of nanopowders alone, 100 µL of the sample solution and 100 µL of the bacterial suspension were added per well. Control wells contained only bacterial culture without any test compound. Plates were incubated at 36 ± 1 °C for 18–24 h. After incubation, bactericidal activity was assessed by preparing ten-fold serial dilutions from each well and plating them onto Mueller–Hinton solid media, and the resulting colony-forming units (CFUs) were counted following further incubation.

The MBC was defined as the lowest concentration of the sample that resulted in a ≥99.9% reduction in colony-forming units per milliliter (CFU/mL), signifying a bactericidal effect. Meanwhile, the MIC was identified as the lowest concentrations of the samples that inhibited the growth of the bacteria or yeast. The concentration of surviving treated microorganisms was calculated using the formula

CFU/mL = (number of colonies × dilution factor)/volume of the inoculum sample

### 4.4. Activated Chemiluminescence Assay

The final active concentration of the newly synthesized nanogel applied was 1 mg/mL (dissolved in deionized twice-distilled water). This is considered very high to demonstrate significant effects. The chemiluminescent signal was compared to that of a control reaction without applied nanomaterial (blank control). The effect of the new material on the kinetics of free radical generation and oxidation was tested ex vivo, at pH 7.4—physiological,—and at pH 8.5—optimal,—at 25 °C by the chemiluminescent method activated by lucigenin in the following chemical model systems [35,69]:

- Fenton’s system: H_2_O_2_–FeSO_4_-generating hydroxyl (^.^OH) and hydroperoxyl (^.^OOH) radicals;
- System containing hydrogen peroxide (H_2_O_2_);
- (NAD.H–PhMS (phenazine methosulfate)) system generating superoxide radicals (O_2_^−.^);
- All experimental data were statistically processed by MSOffice Pro 2021 and Origin Pro 8; the significant effects were calculated as quantum yields, which are integral values describing the total light, emitted from the reaction;
- Fenton’s system: 0.2 mol sodium hydrogen phosphate buffer with the chosen pH, Fenton’s reagent (FeSO_4_ (5 × 10^−4^ mol)—H_2_O_2_ (1.5%)), and lucigenin (10^−4^ mol); the interaction follows the scheme below, producing various ROS:
  (1) Fe^2+^ + H_2_O_2_ → Fe^3+^ + ^.^OH + ^−^OH
  (2) Fe^3+^ + H_2_O_2_ → Fe^2+^ + ·OOH + H^+^
- System containing hydrogen peroxide (H_2_O_2_): 0.2 mol sodium hydrogen phosphate buffer with the chosen pH, H_2_O_2_ (1.5%), and lucigenin (10^−4^ mol); in this chemical model system, hydrogen peroxide reacts as an oxidant and a ROS.
- NAD.H–PhMS: 0.2 mol sodium hydrogen phosphate buffer with the chosen pH, NAD.H (10^−4^ mol), phenazine methosulfate (10^−6^ mol), and lucigenin (10^−4^ mol); the interaction is following the scheme below, producing superoxide radicals:
  (1) PhMS + NAD.H + H^+^→ PhMS.H_2_ + NAD^+^
  (2) PhMS.H_2_ + PhMS → 2 PhMS.H^.^
  (3) PhMS.H^.^ + O_2_ → PhMS + O_2_^−.^ + H^+^

All reactions were monitored for 3 min, every 3 s, performed in triplicate, with *p* ≤ 0.05 (Figures S1–S3). All experiments were conducted by LUMIstar Omega (BMG Labtech GmbH, Ortenberg, Germany, 2020). The tested nanogel was sonicated for at least 60 min before application.

## Figures and Tables

**Table 1:** Observed cut-off and calculated bandgap values of selected samples.

Samples	Gels		Heat-Treated Samples (600 °C)	
	Cut-Off (nm)	Eg (eV)	Cut-Off (nm)	Eg (eV)
TiO_2_ from Ti(IV) butoxide	389.71	3.18	412.5	3.01
80TiO_2_/10TeO_2_/10Nb_2_O_5_	363.2	3.41	454.2	2.73

## Data Availability

Data are contained within the article.

## Supplementary Materials

The following supporting information can be downloaded at https://www.mdpi.com/article/10.3390/gels11090716/s1, Figure S1. Effect of Nb_2_O_5_-containing nanosized powders on chemiluminescence, presented as Reference Luminescent Units (RLUs), in a system for the generation of ^·^OH and ^·^OOH radicals at pH 8.5 (a) and pH 7.4 (b), Figure S2. Effect of Nb_2_O_5_-containing nanosized powders on chemiluminescence, presented as Reference Luminescent Units (RLU), with oxidant H_2_O_2_, Figure S3. Effect of Nb_2_O_5_ containing nanosized powders on chemiluminescence, presented as Reference Luminescent Units (RLU), in a system for the generation of O_2_^−^· radicals at pH 8.5 (a) and pH 7.4 (b).
