# Supplementary material for: The Antimicrobial Effect and ROS Redox Activity of Nb2O5-Containing Powders Obtained by the Sol–Gel Method
Source: Gels. 2025 Sep 7;11(9):716. doi: 10.3390/gels11090716 (PMC12469671; doi:10.3390/gels11090716)
Supplement: Supplementary file 1 [file gels-11-00716-s001.zip › gels-3834999-supplementary.pdf]

Supplementary material for:
Antimicrobial Effect and ROS-Redox Activity of Nb_2O_5–Containing Powders Obtained by the Sol-Gel Method

Kalina Ivanova[1,4*], Elitsa Pavlova[2,5], Iliana Ivanova[3], Albena Bachvarova-Nedelcheva[1,4*]

[1] Institute of General and Inorganic Chemistry, Bulgarian Academy of Sciences, Acad. G. Bonchev Str., Bl. 11, 1113 Sofia, Bulgaria; albenadb@svr.igic.bas.bg

[2] Faculty of Physics, Sofia University "St. Kliment Ohridski", 5 James Bourchier Blvd., 1164 Sofia, Bulgaria; elli_pavlova@abv.bg

[3] Faculty of Biology, Sofia University "St. Kliment Ohridski", 8 Dragan Tsankov Blvd., 1164 Sofia, Bulgaria; iaivanova@biofac.uni-sofia.bg

[4] National Centre of Excellence Mechatronics and Clean Technologies, 8 bul., Kl. Ohridski, 1756, Sofia, Bulgaria

[5] Center of Competence "Clean Technologies for Sustainable Environment—Water, Waste, Energy for Circular Economy", 1000 Sofia, Bulgaria

* Correspondence: kalina@svr.igic.bas.bg; albenadb@svr.igic.bas.bg

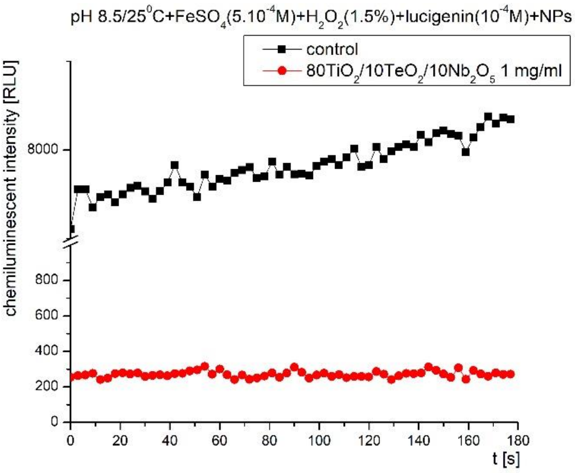

(a)

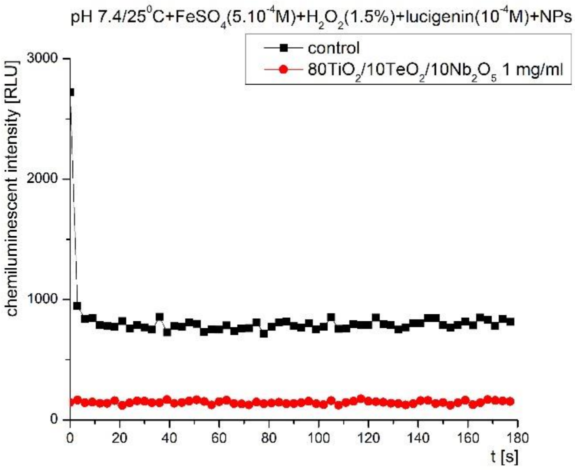

(b)

Figure S1. Effect of Nb_2O_5 containing nanosized powders on chemiluminescence, presented as Reference Luminescent Units (RLU), in a system for the generation of ·OH and ·OOH radicals, at pH 8.5 (**a**) and pH 7.4 (**b**).

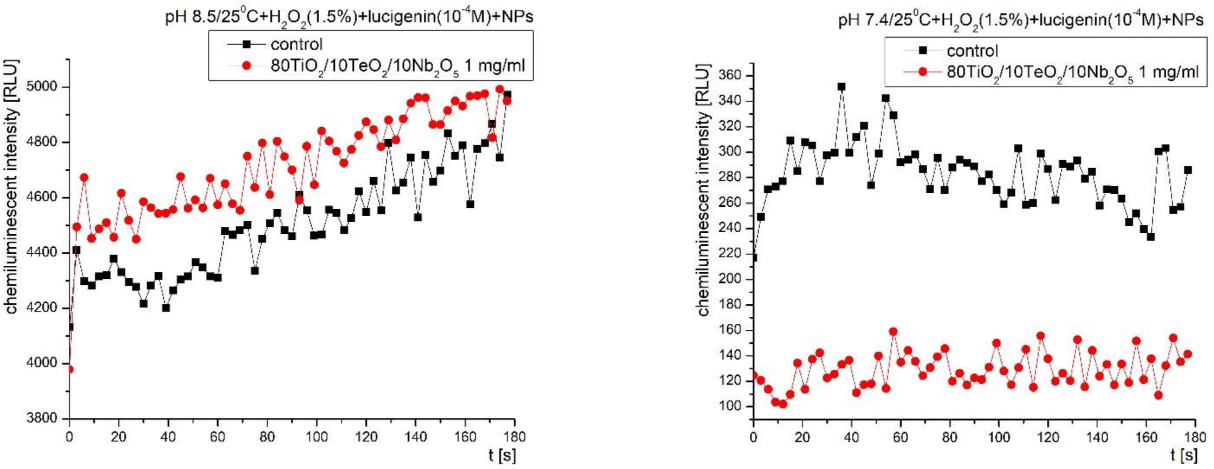

(a) (b)

Figure S2. Effect of Nb_2O_5 containing nanosized powders on chemiluminescence, presented as Reference Luminescent Units (RLU), with oxidant H_2O_2, at pH 8.5 (**a**) and pH 7.4 (**b**).

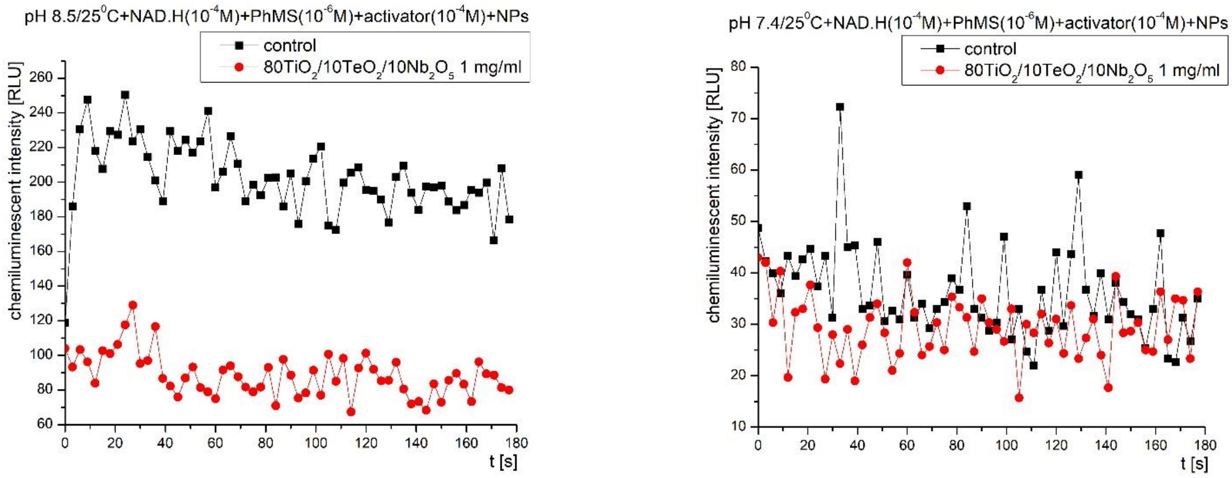

(a) (b)

Figure S3. Effect of Nb_2O_5 containing nanosized powders on chemiluminescence, presented as Reference Luminescent Units (RLU), in a system for the generation of $O_2^{-}\cdot$ radicals at pH 8.5 (**a**) and pH 7.4 (**b**).
